# Immunotherapy in Cancer: A Combat between Tumors and the Immune System; You Win Some, You Lose Some

**DOI:** 10.3389/fimmu.2015.00127

**Published:** 2015-03-26

**Authors:** Florencia Paula Madorsky Rowdo, Antonela Baron, Mariela Urrutia, José Mordoh

**Affiliations:** ^1^Laboratorio de Cancerología, Fundación Instituto Leloir – IIBBA-CONICET, Buenos Aires, Argentina; ^2^Centro de Investigaciones Oncológicas, Fundación Cáncer and Instituto Alexander Fleming, Buenos Aires, Argentina

**Keywords:** cancer immunotherapy, CTLA-4, PD-1, monoclonal antibodies, vaccines, melanoma

## Abstract

Cancer immunotherapy has emerged as a treatment modality, mainly as the result of discoveries in the immune response regulation, including mechanisms that turn off immune responses. Immunogenic cutaneous melanoma is a canonical model for therapeutic immunotherapy studies. “Passive” immunotherapy with monoclonal antibodies (mAbs) has outpaced “active” immunotherapy with anti-tumor vaccines, and mAbs that antagonize the *off* responses have been recently introduced in clinical practice. Despite these recent successes, many unresolved practical and theoretical questions remain. Notably unknown are the identity of the lymphocytes that eliminate tumor cells, which white cells enter into tumors, through which endothelium, in what order, and how they perform their task. The parameters of size and location that could be used to determine in which tumors the immune response may be sufficient to eradicate the tumor are yet unknown. Immunotherapy has been so far more efficient to treat solid and hematologic tumors located outside the central nervous system, than primary brain tumors and brain metastases. In contrast to recent advances with mAbs, anti-tumor vaccine development has been lagging behind. The multiplicity of antigens that must be targeted to achieve significant clinical response is partially responsible for this lag, especially in melanoma, one of the most mutated tumors. Further hampering vaccination results is the fact that tumor elimination by the immune system is the result of a race between tumors with different growth rates and the relatively slow development of the adaptive immune response. The enhancement of the native arm of the immune response or the administration of targeted chemotherapy to slow tumor development, are approaches that should be studied. Finally, criteria used to analyze patient response to immunotherapeutic treatments must be perfected, and the patient populations that could benefit the most from this approach must be better defined.

## Introduction

After decades of cautious approach, immunotherapy has arrived as a cancer treatment. This is best demonstrated by the therapeutic benefit conferred by monoclonal antibodies (mAbs) targeting the immune checkpoints CTLA-4/CD80/CD86 and PD-1/PD-L1 in metastatic cutaneous melanoma (CM). Anti-tumor effects are accompanied by autoimmunity, an affordable price for the clinical responses obtained. However, there is still room for improvement of this therapeutic approach. Important questions still remain, which, if adequately answered, may foster this new field. In this review, we continue our previous discussion (1), and delve into several such points. We shall review, although not exhaustively, some recent findings about the immunoregulatory molecules CTLA-4 and PD-1, with special emphasis on their role in CM. We shall also review the most important clinical trials performed with their respective mAbs. In the course of this review, we shall address several important points, such as:

(1) Is the presence of lymphocytic infiltrate in human tumors necessary prior to anti-CTLA-4 or anti-PD-1 mAb administration?
(2) What are the mechanisms that activate CD8+ lymphocytes as putative effectors of the clinical response obtained in CM patients?
(3) What are the specificities of these anti-tumor lymphocytes?
(4) What mechanisms do lymphocytes use to enter tumors, and how can they be improved?
(5) Can tumor lymphocytic infiltrate be increased by prior vaccination?

## CTLA-4: Basic Knowledge

Cytotoxic T-lymphocyte antigen-4, CTLA-4 (CD152), is a type I transmembrane glycoprotein that presents homology to CD28 and down-regulates T-cell activation, playing a key role in the regulation of immune homeostasis. CTLA-4 surface expression is induced in activated effector T cells (Teff cells) (2). It is constitutively expressed in high levels in CD4+CD25+ regulatory T cells (Treg cells) (3) and binds with higher affinity than CD28 to the costimulatory molecules CD80 (B7.1) and CD86 (B7.2) expressed by antigen-presenting cells (APC). Different humanized mAbs targeting CTLA-4 have been developed, among them are Ipilimumab, an IgG1 mAb, and Tremelimumab, an IgG2a mAb.

Although anti-CTLA-4 Ipilimumab has been approved for use in metastatic CM patients, as it leads to improved overall survival (OS), its mechanisms of action are not completely understood. Whether anti-CTLA-4 antibody acts directly in *cis* on the Teff cell compartment, by blocking inhibitory signals without cellular interaction with other lymphocytes (4, 5), or if it behaves indirectly in *trans*, through Treg cell depletion or limitation of their immunosuppressive function (6), is not yet clear. Pre-clinical studies in the B16/BL6 murine melanoma model have demonstrated that the Gvax plus anti-CTLA-4 blockade increased the number of tumor-infiltrating lymphocytes (TILs), attaining 1.4 × 10^6^ TIL per gram of tumor tissue (approximately 10^9^ tumor cells) (7), though it is difficult to explain how this effector/target ratio (1/1000) could achieve tumor regression. Curran et al. demonstrated in the same experimental model that treatment with anti-CTLA-4 or anti-PD-1 increased the CD8+ Teff/Treg ratio around 10-fold, that the effector/tumor-cell ratio was about 1–2/1000, and that only when both mAbs were combined with a cellular vaccine, cures were observed (8). In a murine prostate cancer model, Waitz et al. observed that cryoablation of tumors plus anti-CTLA-4 increased the CD8+ and CD4+ infiltration of secondary tumors, attaining infiltration of 1 CD4+ and CD8+ lymphocyte per 1000 tumor cells (9). These results could be achieved by *cis* and/or *trans* mechanisms. However, other work in the B16/BL6 murine melanoma model by Simpson et al. reported a previously undescribed mechanism of action for the anti-CTLA-4 mAb, which involved an antibody-dependent cellular cytotoxicity (ADCC)-mediated depletion of intra-tumoral Treg cells by FcRIV-expressing macrophages. This would lead to an increase in the intra-tumoral Teff/Treg ratio (10) and suggested a predominant *trans* mechanism. Recent pre-clinical studies highlight the roles of Fcγ receptors (FcγR) and the tumor microenvironment in the activity of different immunomodulatory antibodies (11), including anti-CTLA-4. The ADCC-mediated mAb effect was also described for an anti-GITR antibody (GITR: glucocorticoid-induced TNFR-related protein) (12) and for an anti-OX40 antibody (13). Consistent with these observations, different anti-tumoral efficiencies were detected between different anti-CTLA-4 antibody isotypes in mouse models, the most efficient being IgG2a, a strong binder of activating FcγR (14). However, if ADCC was the mechanism of action of Treg cell lysis, it is unclear why CD8+ lymphocytes were not also depleted, although, Treg cells express higher levels of CTLA-4. Pointing to a concurrent *cis* mechanism of action, experimental evidence in CTLA-4^−/−^ mice carrying human CTLA-4 suggested that anti-CTLA-4 mAb would need to bind to both Teff and Treg cells to produce full tumor protection (15).

Turning now to the human setting, if a favorable balance of the Teff/Treg ratio appears necessary to induce anti-tumor responses, a relevant matter is how important is the actual number of intra-tumoral lymphocytes before and after therapy, and if the lymphocytic infiltrates are within the tumor (brisk) or peripheral (non-brisk). With respect to the lymphocytes present before therapy, the question is best addressed in primary tumors and visceral metastases, since lymphocytes-infiltrating lymph node metastases are difficult to differentiate from residing lymphocytes, hence their name tumor-associated lymphocytes (TAL) (16). Whereas the presence of TIL in primary tumors with a Breslow index between 1.7 and 6.0 mm is associated with better prognosis, the prognostic evidence regarding the presence of TIL in metastases is less clear [see Oble et al. for a review (17)]. Hakansson et al. have performed fine-needle-aspiration in CM metastatic patients and observed that metastasis with >2% CD4+ lymphocytes responded better to biochemotherapy than patients with <2% CD4+ lymphocytes (18). Anyhow, the number of lymphocytes relative to tumor cells appears to be low (around 1/10^3^), especially if one takes into account the low affinity of the TCR/MHC I-peptide complex (10^−4^–10^−5^ M) and that the estimated number of lytic cycles per cytotoxic T-lymphocyte is low (19). Therefore, it is probable that the number of “spontaneously occurring” lymphocytes in a tumor should be dramatically augmented to attain a meaningful clinical response. A possible factor that could augment TIL within tumors derives from reports suggesting that CTLA-4 blockade increases T-cell motility (20–22). In a study using intravital microscopy in the mouse model B16/BL6, Pentcheva-Hoang et al. analyzed the motility of reporter pmel-1 T cells and reported that chronic anti-CTLA-4 treatment increased pmel-1 T-cell velocity in tumors and in tumor-draining lymph nodes, whereas acute CTLA-4 blockade increased pmel-1 T-cell velocity exclusively in tumor-draining lymph nodes (22). Whether this phenomenon actually favors the immune response is subject to debate. Increased T-cell motility could favor T-cell scanning, mobilize T cells from unproductive interactions with APC, and increase T-cell infiltration into tumors, which would be extremely advantageous. On the other hand, increased T-cell motility could be detrimental to the immune response by preventing efficient TCR–MHC-I_APC_ or TCR–MHC-I_tumor_ cell interactions. In the clinic, a direct correlation between lymphocytic infiltration and prognosis has not been clearly established. The quantitative distinction between brisk lymphocytic infiltrates, which would be capable of cytolytic activity, and peripheral infiltrates, which would act as a dissemination barrier, is generally not described. The analysis of biopsies of metastatic CM lesions before and after Gvax and Ipilimumab treatment, showed low numbers of CD8+ and Treg lymphocytes in pre-treatment biopsies and dense CD8+ cell infiltration after treatment. However, the low number of infiltrating Treg cells would suggest that intra-tumoral Treg cell lysis is not the main target of Ipilimumab. However, tumor necrosis did correlate with higher infiltrating CD8+ T/Treg cell ratios (23). Evidence from bladder and prostate cancer patients also suggests that anti-CTLA-4 treatment leads to an increased intra-tumoral Teff/Treg ratio (24, 25). The origin of the CD8+ lymphocytes that infiltrate tumors is uncertain. CD8+ cells could derive from the CTLA-4 blockade in central lymphoid organs in cancer patients, since it could promote T-cell proliferation and lead to increased T-cell infiltration in most patients (26, 27). CTLA-4 blockade with Tremelimumab led to a diversification of the peripheral TCR repertoire (28), an observation that was reiterated in a separate study in patients treated with Ipilimumab (29). Additionally, the maintenance of high frequency TCR clones was associated with improved clinical outcome. Recent evidence by Kvistborg et al. suggests that CTLA-4 blockade broadens CM-specific T-cell response (30). These authors analyzed HLA-A*0201-restricted epitope recognition by CD8+ T cells in CM patients before and after Ipilimumab, and reported that anti-CTLA-4 induced CD8+ T-cell clones absent before treatment, although pre-existing clones were not significantly boosted. However, not every intra-tumoral CD8+ cell was functional or directed against tumor antigens, since tumor infiltration by T cells in patients treated with Tremelimumab did not correlate with clinical responses (27). This suggests that resistance to CTLA-4 blockade could depend on other immunosuppressive mechanisms displayed by the tumor. Thus, the current understanding is as follows: experimental data suggest that successful anti-CTLA-4 treatment would require abundant Treg cells, CD8+ lymphocytes, and NK cells and/or macrophages within the tumor; although it is not yet known in what quantity or if they should be present as brisk or peritumoral infiltrates. It is likely that CD8+ lymphocytes will need to be expanded by treatment, although the optimal time span is unknown. Once Ipilimumab is administered, there are two main possibilities: (i) CD8+ cell proliferation would take place in lymphoid organs, and higher numbers of CD8+ cells with increased motility would migrate into tumors; (ii) Ipilimumab would mediate the ADCC of Treg cells through the FcγR of residing macrophages and/or NK cells; CD8+ cells would be relieved from Treg cell downregulation, and would kill tumor cells. However, the latter possibility does not take into account that the number of Treg cells, CD8+ lymphocytes, macrophages, and NK cells in many metastases are insufficient before treatment begins (18).

## Clinical Response to Anti-CTLA-4 Treatment

In a randomized Phase III trial for 676 HLA-A*0201 CM patients with unresectable Stage III or Stage IV disease (31), previously treated patients and whose disease had recurred, received, in a 3:1:1 ratio, Ipilimumab (3 mg/kg every 3 weeks for a total of four doses) plus gp100 peptide vaccine (403 patients); Ipilimumab alone (137 patients); or gp100 alone (136 patients). Around 60% of patients treated with Ipilimumab presented immune-related adverse events (irAE), mainly gastrointestinal (diarrhea, colitis), dermatologic (pruritus, rash), and endocrine (hypothyroidism, hypopituitarism); 10–15% were grade 3–4 toxicities. The median OS for the three patient groups were 10.0, 10.1, and 6.4 months, respectively. A 3.5-month gain in OS was obtained with Ipilimumab, and the drug was approved for the treatment of metastatic CM (Table 1). The gp100 vaccine was composed of two gp100 peptides, 209–217 and 280–288, in four doses of 1 mg each, emulsified in Montanide ISA-51. Because two of the study arms administered gp100 peptides that are presented by the HLA-A*0201 haplotype, this study was performed exclusively on HLA-A*0201 patients. Whether HLA status was relevant to Ipilimumab efficacy remained unanswered, until a retrospective analysis of Ipilimumab efficacy in populations with HLA-A*0201-positive and negative haplotypes yielded similar clinical results (32). These results strongly suggest that CD8+ lymphocytes are active against melanoma cells, regardless of HLA-A*0201 haplotype.

**Table 1:** **Selected clinical trials with anti-checkpoint mAbs**.

Patients	Phase	Treatment	*n*	Study groups	Results	Toxicity	Reference
**ANTI-CTLA-4**							
Melanoma HLA-A*0201-positive unresectable stage III or IV previously treated	III	Ipilimumab	676	Ipilimumab plus gp100	OS 10.0 months groups I versus 10.1 months group II versus 6.4 months group III	10–15% irAE grades 3–4	(31)
		gp100 vaccine		Ipilimumab	
				gp100	
Melanoma unresectable stage III or IV previously untreated	III	Ipilimumab DTIC	502	Ipilimumab (10 mg/kg i.v.) plus DTIC (850 mg/m^2^) DTIC (850 mg/m^2^) plus placebo	OS 11.2 months group I versus 9.1 months group II	56.3% grades 3–4 group I, 27.5% grades 3–4 group II	(33)
**ANTI-PD-1**							
Melanoma unresectable stage III or IV	Ib	Lambrolizumab	135	10 mg/kg every 2–3 week	OR 38%	13% grades 3–4 irAEs	(34)
				2 mg/kg every 3 week	
Melanoma unresectable stage III or IV BRAF wild-type previously untreated	III	Nivolumab DTIC	418	Nivolumab 3 mg/kg every 2 week DTIC 1000 mg/m^2^ every 3 week	OS (1 year) 72.9% group I versus 42.1% group II	11.7% grades 3–4 group I, 17.6% grades 3–4 group II	(35)
Refractory or relapsed Hodgkin’s lymphoma pre-treated	I	Nivolumab	23	1–3 mg/kg dose escalation cohort, expansion cohort 3 mg/kg 1, 4 weeks and then every 2 week	OR 87%, PR 70%, CR 17%	22% grade 3	(36)
Advanced melanoma, NSCLC, prostate cancer, renal-cell cancer, CRC	I	Nivolumab	296	1, 3, and 10 mg/kg	OR 18–28%	14% grades 3–4	(37)
**ANTI-CTLA-4 + ANTI-PD-1**							
Melanoma unresectable stage III or IV previously treated with Ipilimumab	I	Nivolumab Ipilimumab	53	Concurrent regimen	OR 40%	53% grades 3 or 4	(38)

*irAEs, immune-related adverse events; OS, overall survival; OR, overall response; CR, complete response; PR, partial response*.

Another randomized Phase III Ipilimumab study was performed for 502 patients with previously untreated metastatic CM. Ipilimumab in combination with dacarbazine (DTIC) was compared to DTIC plus placebo; the median OS in the first group was 11.2 versus 9.1 months in the DTIC plus placebo group (33) (Table 1). Remarkably, Ipilimumab treatment may take months to induce tumor remission, indicating that it acts by different mechanisms of action than most chemotherapeutic agents.

Prior to Ipilimumab approval by the FDA, an anti-CTLA-4 IgG2 mAb (Tremelimumab) was developed by Pfizer (CP-675,026) and assayed in a Phase I clinical trial on 39 patients, of whom 29 had CM, in doses ranging between 0.01 and 15.0 mg/kg. The primary toxicities were dermatitis and diarrhea (39). In that trial, 2/29 CM patients had partial responses (PRs) and 2/29 had complete responses (CRs). The evidence remains unclear as to the mechanism of anti-tumor action of Tremelimumab. It should be taken into account that the interpretation of results is often complicated by differences in biopsy timing. Thus, in a report of a Phase I trial of mAb CP-675,026 (39), immunohistochemical (IHC) analysis of a residual tumor mass after treatment revealed inflammatory infiltration by CD15-positive macrophages, but no CD3+ lymphocyte infiltration, perhaps suggesting that a long-term response may also involve macrophages. Huang et al. (27) demonstrated in a Phase II study, which compared paired biopsies before and after treatment that 14/18 evaluable CM patients treated with Tremelimumab had increased CD8+ lymphocyte infiltration, but this infiltration was not correlated with clinical responses. Instead, they found an increase of Treg cells in responding patients. It is interesting to note that in that study, intra-tumoral CD8+/Ki67+ cells were scarce, even in a patient with CR, suggesting that the CD8+ pool expansion took place outside the tumor. A Phase III randomized trial (A3671009) comparing Tremelimumab with DTIC in 630 CM patients was halted in 2008 because Tremelimumab failed to improve DTIC outcomes. The discrepancy between the results for Ipilimumab and Tremelimumab is difficult to explain; possible reasons are the different affinities of mAbs for CTLA-4, or the different isotypes of the mAbs utilized, IgG1 and IgG2a, respectively, since they have different capacities for binding complement and FcγR (40).

So far, the activity of anti-checkpoint mAbs has been detected in peripheral tumors. The possible activity of these agents in primary brain tumors is largely unexplored, one of the predicted obstacles being the poor permeation of circulating blood cells and large molecules through the hematoencephalic barrier. With respect to brain metastases of several tumors, which constitute a huge medical problem, in most clinical trials using anti-checkpoint mAbs, patients with brain metastases have been excluded. Nevertheless, the activity of Ipilimumab in 72 melanoma patients with brain metastases was assayed in an open-label, Phase II trial (41). The results suggested that Ipilimumab may have some activity in patients with small, asymptomatic metastases. Due to the large number of tumors that metastasize into the central nervous system, this field deserves increased attention.

In conclusion, Ipilimumab appears to act on tumor cells indirectly, through the activation of the immune system, a mechanism of action consistent with the irAE observed. As to the relevant antigens that mediate tumor regression, the fact that gp100 vaccine added no benefit to Ipilimumab is not surprising, since Aris et al. reported that in most CM, almost half of proliferating cells do not express melanocyte differentiation antigens (MDAs), and that MDA expression has considerable plasticity (42). Also, Chandran et al. recently found that isolation of CTL clones specific to MART-1 and gp100, although exerting powerful cytolytic activity *in vitro*, did not induce important remissions when injected to melanoma patients (43). In an interesting turnabout, Snyder et al. recently reported the results of an exomic analysis of responder versus non-responder patients treated with Ipilimumab and Tremelimumab (44). They found that the high mutation frequency in CM (0.5– >100 mutations per megabase) creates neoantigens with epitopes similar to “foreign,” probably infectious, epitopes. That epitopic similarity would determine that Teff lymphocytes directed against those foreign epitopes would then be able to recognize mutated tumor antigens. Therefore, higher mutation frequencies could be a favorable condition, although not determinant, for a good clinical response in anti-immune checkpoint mAb therapy. In an experimental murine model of a highly mutated methylcholanthrene-induced sarcoma, Gubin et al. have also found specific mutations that generate neoepitopes, which may be identified and used to generate long peptidic vaccines that alone or combined with antibodies anti-checkpoint CTLA-4 and/or PD-1 could afford tumor protection and elimination (45).

## The Biology of the PD-1 Pathway

The programed cell death protein-1, PD-1 (CD279), is an inhibitory receptor of the extended CD28 family of T-cell regulators. Besides being expressed in activated T cells, PD-1 is also expressed in B cells and monocytes. PD-1 binds PD-L1 (B7-H1/CD274) (46) and PD-L2 (B7-H2/CD273) (47). PD-L1 is expressed in T cells, B cells, macrophages, dendritic cells (DCs), and some non-immune cells, and is upregulated after activation. PD-L2 is more tightly regulated and is primarily expressed on activated macrophages and DCs. PD-1 is expressed on the surface membrane of activated T cells, and its main role is to limit autoimmunity and T-cell activity in peripheral tissues during an inflammatory response to infection (48–50). T-cell activation induces PD-1 expression, and PD-L1 binding leads to the inhibition of T-cell activation and effector function. This inhibition is mediated by the recruitment of phosphatases to the immune synapsis that dephosphorylate molecules related to TCR signaling (51).

PD-L1 is not detectable in most normal, non-inflamed tissues (52–54), but is highly expressed in several human tumors including CM (55). Since it is minimally expressed in the adjacent normal tissue, it has been suggested that PD-L1 has a role in attenuating anti-tumor immune responses (52, 53). IFN I and II upregulate PD-L1 expression by tumor cells (52, 56), thus promoting the apoptosis of antigen-specific T cells *in vitro*, and it has also been suggested in a murine model that PD-L1+ tumors deleted activated T cells *in vivo* (52). Additionally, PD-L1 has been implicated in tumoral antiapoptotic activity, and both mechanisms would favor tumor development (57–59). In addition to PD-L1/PD-1 recognition, an unexpected PD-L1/CD80 interaction was detected (60), whereby CD80 expressed on T cells took on a receptor role and delivered inhibitory signals in response to PD-L1 binding (61, 62). In CM, PD-L1 expression was correlated with TILs (63–65). Taube et al. observed that 98% of PD-L1+ tumors were associated with TILs. They proposed that TILs trigger their own inhibition by secreting IFN-γ, among other cytokines, that drive tumor PD-L1 expression (63). Clinically, PD-L1 expression levels on tumors correlate with poor clinical outcome for patients with several types of cancer, including CM (66–71).

In a murine model, PD-1 was highly expressed on Treg cells and promoted their function and proliferation in the presence of the ligand (72). As many tumors are infiltrated or surrounded by host immune suppressor Treg cells, blockade of PD-1 may upmodulate anti-tumor immune response by decreasing intra-tumoral Treg cell number or action (73). Comparing the two inhibitory receptors described in this review, PD-1 acts in the peripheral tissues and in tumors, regulating effector T-cell activity, whereas CTLA-4 acts in the lymph nodes, regulating T-cell activation (73). The narrow therapeutic window for anti-CTLA-4 therapy can be partially attributed to the fact that CTLA-4 ligands are not expressed in tumor cells. Contrarily, PD-L1/PD-1 would interact selectively in the tumor microenvironment (63).

All of the previously mentioned data strongly support a central role for PD-1 and/or its ligands in tumor immune escape. Several mAbs were developed against PD-1: (a) Nivolumab, also known as BMS-936558 or ONO-4538; (b) Pembrolizumab formerly Lambrolizumab, also known as MK-3475; (c) Pidilizumab (CT-011); and anti-PD-L1 (BMS-936559; MEDI4736; MPDL33280A). PD-1 blockade has been shown to enhance T-cell responses and presented anti-tumoral activity in pre-clinical models (52, 57, 74).

## Clinical Responses to Anti-PD-1 and Anti-PD-L1 Treatments

BMS-936558/Nivolumab is a fully humanized mAb, IgG4 (kappa) that binds PD-1. Nivolumab was assayed in a Phase I trial involving 296 patients with advanced solid tumors: CM, non-small-cell lung cancer, castration-resistant prostate cancer, renal-cell cancer, and colorectal cancer. The doses assayed varied between 0.1 and 10.0 mg/kg i.v., every 2 weeks, for periods lasting more than 1 year. PD-L1 expression was assessed by IHC, and tumors with >5% cells with membrane-expressed PD-L1 in any lesion were deemed positive. Of the 106 patients with CM, 26 objective responses (ORs) (24.5%) were observed, many of which achieved stable disease. When tumors were stratified according to PD-L1 status, 36% OR were observed in PD-L1-positive patients versus 0% OR in PD-L1-negative patients. Thus, PD-L1 expression levels would appear to be a good predictive marker for anti-PD-1 therapy. The adverse events reported for this study was about 41%, 6% being grade 3 or 4 serious adverse events (37). Recently, Robert et al. reported the results of a Phase III clinical study in which Nivolumab (3 mg/kg every 2 weeks) or DTIC (1000 mg/m^2^ every 3 weeks) were administered to 418 previously untreated patients with stage III or stage IV unresectable, BRAF wild-type, tumors. At 1 year, the OS was 72.9% for the Nivolumab group versus 42.1% for the DTIC group; the median progression-free survival was 5.1 months for Nivolumab versus 2.2 months for the DTIC group, and the overall response rate was 40.0% for Nivolumab versus 13.9% for the DTIC group (35) (Table 1). In this study, PD-L1 was determined prospectively and was considered positive if >5% of at least 100 cells in any histological section had positive membrane staining. The CR rate was 7.6% in the Nivolumab group versus 1.0% in the DTIC group. In contrast to previous results from this mAb, the status of PD-L1 did not affect the response to Nivolumab.

Hamid et al. reported the results of a Phase I clinical study with another anti-PD-1 mAb, Lambrolizumab/Pembrolizumab (MK-3475, Merck Sharp and Dome) on 135 patients with advanced CM, half of whom had received prior Ipilimumab treatment (Table 1). Lambrolizumab is an IgG4 humanized mAb, whose variable region derives from a high-affinity murine mAb (dissociation constant: 29 pM). The doses utilized were 2 or 10 mg/kg, every 2–3 weeks; the half-life of serum mAb was 2–3 weeks. The response rate was 38% and the overall median progression-free survival among the 135 patients was longer than 7 months. Toxic effects were observed in 79% of the patients, but only 13% were grade 3 or 4 toxicities. Patients were not selected on the basis of PD-L1 expression by tumor cells (34). Retrospective analysis of PD-L1 expression was performed in this group of patients; PD-L1-positive patients had a 53% response rate whereas only 6% PD-L1-negative patients responded (*P* = 0.004). However, durable clinical responses were observed in both PD-L1 positive and negative patients (75). This study also supports a correlation between PD-L1 expression and response to anti-PD-1, although the lower limit of PD-L1 expression was set to 1% of tumoral or stromal cells (see below).

Another study aimed to inhibit PD-L1 through the administration of BMS-936559, a humanized IgG4 mAb that binds PD-L1 and inhibits its binding to PD-1 and CD80. In this Phase I clinical study, BMS-936559 was administered to patients with a variety of solid tumors at doses ranging from 0.3 to 10 mg/kg, every 2 weeks, in 6-week cycles. ORs ranged from 6 to 17% in different tumors, and prolonged stabilization of the disease was observed in 12–41% of the patients at 24 weeks (76).

A combination of Nivolumab and Ipilimumab was also assayed in CM patients (38), and attained an OR rate of 50%. PD-L1 levels in tumor cells were measured and deemed positive when ≥5% of at least 100 tumor cells were positive in any section. In this study, no relationship was found between the level of PD-L1 expression and the responses obtained.

In conclusion, the relationship between PD-L1 expression and the efficacy of the anti-PD-1 mAbs is still unclear. Studies have used different techniques to measure PD-L1: (i) some of them include normal stromal cells in the criteria of positivity, whereas others only measure tumor cells; (ii) some groups fix a lower threshold for PD-L1 positivity at 1% (75), and others at 5% (37, 38). Some studies found a relationship between PD-L1 positivity and clinical response (37, 75), and others have not (35, 38). Furthermore, the precise mechanism of action of anti-PD-1 mAbs is unclear. It is difficult to visualize how the 1–5% of cells that are positive could trigger CD8+ cell reactivity toward the whole tumor by blocking PD-L1/PD-1 interaction with anti-PD-1 mAbs; one would have to assume an “immunological chain reaction” that has not yet been described. Secondly, Nivolumab and Lambrolizumab were designed with an IgG4 isotype to avoid complement or ADCC-mediated lysis of CD8+ cells and the probability of cytokine release syndrome. This would imply that intra-tumoral Treg cells remain unaffected and conflicts with the experimental evidence obtained with anti-CTLA-4 mAbs that demonstrated that elimination of Treg cells was necessary for CD8+ expansion and activity. Four patterns of clinical response have been described in studies using anti-CTLA-4 and anti-PD-1 mAbs: (1) response in baseline lesions by week 12; (2) stable disease with response in some lesions; (3) responses after an increase in the size of some lesions, and (4) reduction of total tumor burden after week 12, with the appearance of new tumor lesions early on (77). We have not found any data describing the cellular composition of the tumor lesions that grew before attaining clinical response. Is it pure tumor growth, or is the increase in size due to tumor infiltration by lymphocytes with inflammation and edema?

Furthermore, the origin of the CD8+ lymphocytes that are presumably the effector cells of the anti-CTLA-4 and anti-PD-1 mAb has yet to be determined. In a paper by Huang et al., only scarce intra-tumoral CD8+/Ki67+ lymphocytes were observed in some Tremelimumab-responder patients, suggesting that expansion of the CD8+ pool took place outside the tumor itself (27). However, Tumeh et al. recently reported that the best predictors for response to anti-PD-1 treatment is a high concentration of CD8+ lymphocytes at the growing border of tumors coexisting with PD-L1 expression by tumor cells; blocking the interaction of the PD-1/PD-L1 axis would trigger IFN-γ release and further expansion of CD8+ lymphocytes within the tumor (78).

Regarding the efficacy of anti-PD-1 mAbs in other tumors, Hodgkin’s lymphoma consists of tumor Reed–Sternberg cells surrounded by an inflammatory infiltrate; tumor cells have an amplification of 9p-24.1 region harboring PD-L1 and PD-L2 loci. Recent results of a Phase I trial on patients with relapsed or refractory Hodgkin’s lymphoma suggest that Nivolumab may be useful, since 87% of the patients achieved CR (17%) or PR (70%) (36). Analysis of Reed–Sternberg cells in pre-treatment biopsies indicated a gain in copy numbers of PD-L1 and PD-L2 ligands. In this study, PD-1 expression by infiltrating T cells was of low intensity, which has been postulated as a favorable predictor to anti-PD-1 treatment (79).

**Figure 1:**
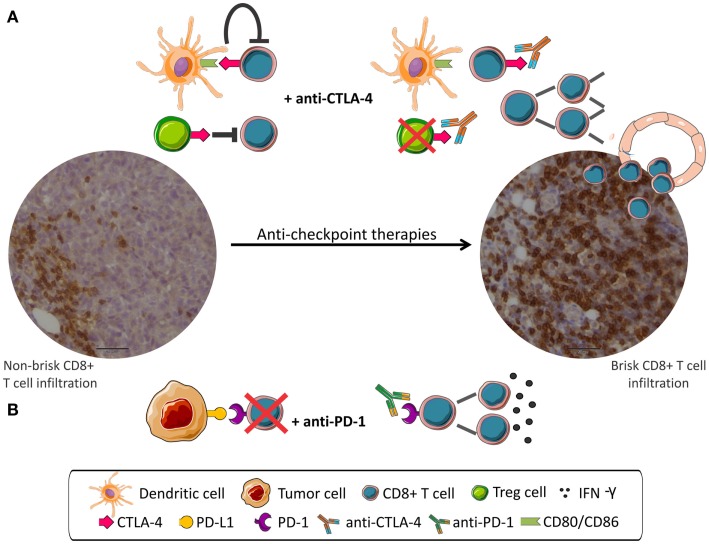
**Anti-checkpoint therapies targeting CTLA-4 or PD-1**. Peripheral CD8 infiltration (left) become tumor invasive (right) after anti-checkpoint treatment. **(A)** CD8+ T cells are inhibited by CTLA-4 signaling and by Treg cells. mAbs anti-CTLA-4 interrupt negative signaling resulting in CD8+ cells proliferation. **(B)** PD-1 expressed in CD8+ cells interacts with PD-L1 expressed in tumor cells and leads to CD8+ inhibition. mAbs anti-PD-1 disrupt negative regulation resulting in activation of CD8+ cells.

A schema depicting the mechanism of action of anti-checkpoint mAbs is shown in Figure 1.

## Cancer Vaccines

Although anti-CTLA-4 and anti-PD-1 mAbs demonstrated remarkable anti-tumor activity in advanced CM patients, attaining overall response rates between 40 and 50%, the fact that CR are a few percent indicates that this therapeutic approach needs improvement. It is possible that the generally scarce CD4+ and CD8+ tumor infiltration could hamper mAb efficacy. In that case, the therapeutic activity of mAbs targeting immune checkpoints could be enhanced by an increase in lymphocytic infiltration before treatment. One possible strategy for this increase is to employ anti-tumor vaccines in the adjuvant setting. For several reasons, though mainly due to the heterogeneity and mutability of CM, we believe that the use of monoantigens as vaccines is doomed to failure. We have developed instead a strategy using the mini-allograft vaccine CSF-470 composed of four lethally irradiated allogeneic CM cell lines combined with BCG and soluble rhGM-CSF as adjuvants. The danger signals provided by allogeneic HLA, BCG, and GM-CSF force the migration of APC and macrophages to the vaccination site, where they would phagocyte tumor antigens, present them in an adequate HLA setting to naïve lymphocytes, either *in situ* or upon migration to the draining lymph nodes. This mini-allograft has been assayed in Phase I clinical trials (80, 81) and is currently being tested against medium-dose IFN-α2b as a post-surgical adjuvant therapy in stages IIB-III CM patients (randomized CASVAC-0401 phase II/III trial; clinicaltrials.gov NCT01729663). Occasional tumor biopsies of recurrent CM showed brisk lymphoid infiltration, mainly composed of CD8+, CD4+, and CD20+ lymphocytes with few Treg cells, and striking evidence of tumor-cell killing by CD8+ and CD4+ lymphocytes (82). Six months after vaccination began, circulating NK cells were significantly increased (83). It will be interesting to assess whether prior vaccination with the allogeneic vaccine CSF-470 improves the efficacy of mAbs targeting immune checkpoints.

## Tumor Vessels and Lymphocyte Infiltration

As previously described, much of the activity related to immunotherapy and cancer takes place within tumors, through the action of a variety of immune cells. However, the factors that govern the arrival of these effectors into tumors remain poorly understood. In recent years, growing evidence shows that several redundant regulatory mechanisms simultaneously produce an immunosuppressive tumor microenvironment, which may limit the effectiveness of immunotherapies and lead to ineffective or suboptimal responses. The limited ability of activated lymphocytes to infiltrate the tumor remains a fundamental problem (84). The tumor vasculature itself, due to numerous structural and functional abnormalities, can represent a great barrier for a successful T-cell tumor infiltration (85, 86). Unlike normal vessels, tumor blood vessels show heterogeneous distribution, tortuosity, dilatation, and fragility; this results in leaky tumor vessels, higher interstitial pressure, heterogeneous permeability, and irregular blood flow (87, 88). The ensuing tumor microenvironment, characterized by interstitial hypertension, hypoxia, and acidosis, may undermine immune cell trafficking, proliferation, and function within the tumor (89). Abnormalities in tumor vasculature result from the imbalance between pro- and anti-angiogenic factors. Angiogenic factors such as vascular endothelial cell growth factors (VEGFs), not only suppress the maturation of DCs (90) and trigger Treg cell proliferation (91), but also inhibit leukocyte–vessel wall interactions by down-regulating vascular adhesion molecules, such as intercellular adhesion molecule-1 (ICAM-1), vascular cell adhesion molecule-1 (VCAM-1), E-selectin, and CD34 (92–94); this phenomenon has been defined as “endothelial cell (EC)-anergy.” Thus, the Teff cells circulating in tumor vessels can hardly interact with ECs, roll through the vascular endothelium, and extravasate into the tumor. Recently, Voron et al. have suggested another immunosuppressive property for VEGF. They proposed that the release of VEGF-A by the tumor enhances the expression of inhibitory immune checkpoint molecules (PD-1, Tim-3, CTLA-4, and Lag-3) on activated CD8+ T cells in tumors (95).

Anti-angiogenic therapies can “*normalize*” many of the structural and functional abnormalities of tumor vasculature, decreasing interstitial fluid pressure, increasing oxygenation, and improving drug penetration into tumors (88). Therefore, the combination of anti-angiogenic drugs with cytotoxic chemotherapy or radiotherapy may ameliorate the final outcome (88, 96). Moreover, pre-clinical studies have suggested that anti-angiogenic therapy could increase tumor-infiltrating T-cell numbers (97–100). In murine models, Li et al. demonstrated that VEGF blockade (by expression of a soluble chimeric VEGF receptor – sVEGFR1/R2) improved the efficacy of a GM-CSF-secreting tumor-cell immunotherapy. They observed a correlation between prolonged survival and a significant increase in the number of activated CD4+ and CD8+ TILs, as well as enhanced apoptosis of Treg cells, which modified the Treg/Teff ratio (97). In murine breast cancer models, Huang et al. found that lower doses of anti-VEGFR2 antibody (DC101) therapy did not significantly change vessel density in tumors, but induced vascular normalization, polarized tumor-associated macrophages (TAMs) from an M2-like (immunosuppressive) to an M1-like phenotype (immunostimulatory), decreased myeloid-derived suppressor cells (MDSCs), and significantly increased tumor-infiltrating CD4+ and CD8+ T cells. Indeed, when combined with a vaccine therapy, DC101 produced a CD8+ T-cell-dependent anti-tumor response (100). In the same line of evidence, Shrimali et al. evaluated the synergism of anti-angiogenic agents with adoptive cell transfer therapy in a murine melanoma model, finding increased infiltration of the adoptively transferred cells into the tumor in combination with anti-VEGF therapy, and to a lesser extent when combined with DC101 antibody (99). Additionally, in a murine model of spontaneous pancreatic carcinoma (RIP1-Tag5), vessel normalization by the deletion of *Rgs5* (RIP1-Tag5XRgs5^−^/^−^) resulted in increased immune effector cell infiltration into tumors and substantially prolonged survival after adoptive CD4+ and CD8+ T cells transfer (98). The evidence that anti-angiogenic agents have the potential to recondition the tumor immune microenvironment toward a more immunosupportive profile (89) promotes their combination with immunotherapy.

## Not all Blood Vessels are Bad

The intra-tumoral development of high endothelium venules (HEVs) and a variety of blood vessels could be considered a marker of good prognosis, since HEV are considered the “gateways for TILs” (101, 102). HEV are specialized post-capillary venules normally found in secondary lymphoid organs (with the exception of spleen), where they support high levels of lymphocyte extravasation from the blood into the lymph nodes (103, 104). The endothelial HEV cells are cuboidal and plump, in contrast with the flat cells of the vascular endothelium. HEV express 6-sulfosialyl Lewis X ligands (L-selectin ligands) on their endoluminal surface, which are recognized by the HEV-specific antibody MECA-79 (105) and mediates the initial capture and rolling interactions of lymphocytes along the vessel walls (103, 106). Therefore, the presence of a large number of lymphocytes attached to their walls is not surprising (103). Although HEV are normally restricted to lymph nodes, HEV-like structures are also found in chronically inflamed non-lymphoid tissues in several inflammatory diseases, including rheumatoid arthritis, inflammatory bowel disease, chronic gastritis, and autoimmune thyroiditis (104, 107). In addition, in an experimental vaccination system, HEV-like structures were found in tertiary lymph node structures at vaccination sites with DC/Apo B16 melanoma cells in the B16/BL6 system (108).

In the last years, the presence of HEV-like structures in tumors was reported for the first time. MECA-79+ vessels were observed by IHC in approximately 60–80% of human primary CM and breast, ovary, lung, and colon carcinomas, but were not detected in normal tissue distant from the tumor site (101). Furthermore, HEV were also observed in human CM metastases (109, 110), although the number of tumor HEV was lower than in primary CM (110). Although the impact of HEV in CM patients is unknown, its occurrence within tumors could represent an important prognostic biomarker. In a retrospective study, breast cancer patients with high densities of tumor HEVs had significantly longer metastasis-free, disease-free, and OS rates (101). In concordance, tumor progression from breast carcinoma *in situ* to invasive carcinoma was accompanied by a reduction in the density of tumor HEVs (111). Interestingly, tumor areas with high HEV density localize specifically with lymphocyte-rich tumor areas, as is seen in lymph nodes. Furthermore, a strong correlation between the density of tumor HEVs and the amount of tumor-infiltrating CD3+ T cells (mainly CD8+ T cells) and CD20+ B cells was found in human breast carcinoma (101) and CM (102). T-lymphocytes were frequently observed extravasating or attached to the luminal surface of tumor HEVs. This evidence strongly suggested that, like HEV in lymph nodes, tumor HEV are actively involved in the recruitment of TILs (101), which could lead to tumor suppression. Nevertheless, tumor HEV could also present a gateway for Treg cell entry into the tumor stroma. In breast cancer patients, a significant increase in the density of tumor-infiltrating Treg cells and CD3+ cells was observed in HEV-high density tumors (111). In human CM, the presence of tumor-infiltrating Treg cells seems to be independent of HEV occurrence, since no significant difference in Treg cell infiltration was observed between samples with low and high HEV densities (102). The incidence of Treg cells in tumor HEVs seems to be more complex; a better understanding of the mechanisms involving Treg cells and tumor HEVs is required, in order to find new opportunities for more effective anti-tumor therapy.

## Combination Therapies

Considering the variety of immunosuppressive mechanisms, the development of combined therapies could be key to enhancing cancer immunotherapy.

Several clinical trials are evaluating the combination of anti-angiogenic agents and immune checkpoint inhibitors. One evaluates the potential synergy between Ipilimumab and Bevacizumab, a VEGF inhibitor, in patients with Stage III/IV CM (NCT01950390). Yuan et al. found that pre-treatment blood levels of VEGF were associated with clinical response to Ipilimumab (112). A phase I study (NCT00790010), comparing patients treated with both drugs or Ipilimumab alone reported changes in the intra-tumoral vascular endothelia and increased trafficking and infiltration of immune cells (CD8+ T cells and CD163+ dendritic macrophages) in tumors (113). There are also ongoing clinical trials evaluating anti-angiogenic therapies combined with anti-PD-1/PD-L1 therapies. A Phase Ib study is evaluating the safety and preliminary efficacy of MPDL3280A (an engineered anti-PD-L1 antibody) in combination with Bevacizumab (NCT01633970); alternatively, the anti PD-1 mAb Nivolumab is being evaluated in combination with Bevacizumab (NCT01454102) as maintenance therapy.

Turning our attention to another important regulatory node, indoleamine 2,3-dioxygenase (IDO) is an enzyme that catalyzes the rate-limiting step in tryptophan degradation to kynurenine. Since tryptophan is not synthesized by mammalian cells and is an essential amino acid for lymphocytes, IDO overexpression and subsequent tryptophan depletion has been proposed as an immunosuppressive factor (114, 115). This phenomenon has been reported in CM cells and in draining lymph nodes. IDO overexpression would correlate with tumor progression and invasiveness (116–118). Pre-clinical studies demonstrated that CTLA-4 blockade synergizes with IDO inhibitors (119), indicating that IDO could play a negative role in anti-CTLA-4 therapy.

Inducible T-cell costimulator (ICOS) is another combinatory target molecule of interest. Expressed by activated T cells, ICOS was identified as a component of the anti-tumoral effects of CTLA-4 blockade (120) and its expression was increased in T cells of treated patients. The combination of anti-CTLA-4 antibody and ICOS activation, which was mediated by tumor vaccines engineered to express the ICOS ligand, enhanced anti-tumor response (121). Recently, Ng Tang et al. suggested that the frequency of ICOS+CD4+ T cells can be used as a pharmacodynamic biomarker for anti-CTLA-4 therapy (122). They reported an increase in ICOS+CD4+ T-cell frequency after anti-CTLA-4 treatment.

## Biomarkers/Gene Signatures

The identification of biomarkers associated with clinical response to immunotherapy remains elusive. Several markers have been proposed as indicators of Ipilimumab response. In CM, an increase in the absolute lymphocyte count after two doses of Ipilimumab was associated with clinical response (123). The presence of NY-ESO-1-specific antibodies and CD8+ T cells also correlated with clinical benefit (124). A recent study suggested that the frequency of circulating MDSC correlated with a worst clinical outcome for CM patients treated with Ipilimumab (125).

As previously discussed, PD-L1 expression, even in a small percentage of tumor cells, is postulated as a predictive marker for anti-tumor response of anti-PD-1 and anti-PD-L1 treatment, but evidence is not conclusive, since published results appear contradictory. It could be assumed as an alternative that high-affinity binding of the mAb to PD-1 would be sufficient to block the negative loop that arrests CD8+ cell proliferation.

Gene expression signatures have been proposed as response predictors for different types of immunotherapy. For example, Harlin et al. performed a gene expression profiling of CM metastases and found two different tumor subsets that could be correlated with the presence or absence of T cells. These observations could be explained by the fact that some tumors express chemokines that mediate the recruitment of activated T cells into metastatic sites, while others do not (126). The absence of chemokine expression in some tumors could represent a barrier to effective immunotherapy, since those tumors would not attract lymphocytes.

Recently, Carretero et al. have shown that, in patients undergoing various forms of immunotherapy, some lesions regress whereas others progress (127). When lesions from the same patient were submitted to genomic analysis, they reported that regression was accompanied by high expression of HLA-I molecules, whereas progressing lesions had lost the HLA-I-presentation genes. Regressions were acute-type rejection, and were accompanied by the expression of genes involved in IFN-mediated antigen presentation and IFN-mediated response (STAT-1/IRF1).

## Conclusion

Recent years have seen dramatic advances that demonstrate the power of the immune system to attack tumors, if the adequate brakes are released. However, several challenges remain. A primary challenge is to establish whether Teff lymphocytes are directed to normal tumor-associated antigens, oncofetal antigens, or neoantigens. Another challenge is to establish a method to increase the number of “dormant” lymphocytes, which, in spite of displaying the adequate TCR, must have their brakes released. A third challenge is to create strategies to increase the ingress of lymphocytes into tumors. Vaccines may be adequate for this purpose, since they could be used in an adjuvant setting, at a time in which metastases have not built a superstructure with many immunosuppressive obstacles to overcome. Finally, it is well established that patches of tumor cells with down-regulated HLA-I expression are found in many tumors, including CM. Perhaps, this is one of the reasons why the number of patients who attain CR with mAbs aiming to CD8+ cell activation is still low. It is likely that the activation of NK cell circuitry would be necessary to effectively target HLA-I^−^ tumor cells.

## Conflict of Interest Statement

The authors declare that the research was conducted in the absence of any commercial or financial relationships that could be construed as a potential conflict of interest.
